# Complete Nucleotide Sequence of Watermelon Chlorotic Stunt Virus Originating from Oman 

**DOI:** 10.3390/v4071169

**Published:** 2012-07-24

**Authors:** Akhtar J. Khan, Sohail Akhtar, Rob W. Briddon, Um Ammara, Abdulrahman M. Al-Matrooshi, Shahid Mansoor

**Affiliations:** 1 Department of Crop Sciences, College of Agricultural and Marine Sciences, Sultan Qaboos University, Al-Khod 123, Sultanate of Oman; Email: msohailakhtar1081@hotmail.com (S.A.); m091875@squ.edu.om (U.A.); matrooshi30@hotmail.com (A.M.A.-M.); 2 National Institute for Biotechnology and Genetic Engineering, Jhang Road, Faisalabad 577, Pakistan; Email: rob.briddon@gmail.com (R.W.B.); shahidmansoor7@gmail.com (S.M.)

**Keywords:** WmCSV, *Begomovirus*, squash, Oman

## Abstract

*Watermelon chlorotic stunt virus* (WmCSV) is a bipartite begomovirus (genus *Begomovirus*, family *Geminiviridae*) that causes economic losses to cucurbits, particularly watermelon, across the Middle East and North Africa. Recently squash (*Cucurbita moschata*) grown in an experimental field in Oman was found to display symptoms such as leaf curling, yellowing and stunting, typical of a begomovirus infection. Sequence analysis of the virus isolated from squash showed 97.6–99.9% nucleotide sequence identity to previously described WmCSV isolates for the DNA A component and 93–98% identity for the DNA B component. *Agrobacterium*-mediated inoculation to *Nicotiana benthamiana* resulted in the development of symptoms fifteen days post inoculation. This is the first bipartite begomovirus identified in Oman. Overall the Oman isolate showed the highest levels of sequence identity to a WmCSV isolate originating from Iran, which was confirmed by phylogenetic analysis. This suggests that WmCSV present in Oman has been introduced from Iran. The significance of this finding is discussed.

## 1. Introduction

Viruses of the family *Geminiviridae* are phytopthathogens with circular, single-stranded (ss) DNA genomes encapsidated within characteristic twinned quasi-icosahedral particles. Geminiviruses have been divided into four genera (*Begomovirus*, *Mastrevirus*, *Curtovirus* and *Topocuvirus*) based on their genome organization, host range and insect vector [1]. Among these the whitefly-transmitted geminiviruses are economically most important and widespread [2]. The genus *Begomovirus* encompasses viruses that are transmitted by the whitefly *Bemisia tabaci* and infect a wide range of dicotyledonous plants in tropical, sub-tropical and increasingly warm temperate regions [3]. Begomoviruses have genomes consisting of either one or two ssDNA components. The two components of bipartite begomoviruses are known as DNA A and DNA B, and both are required for infection of plants. Monopartite begomoviruses (with genomes homologous to the DNA A of the bipartite begomoviruses) are often associated with additional ssDNA satellite molecules known as alphasatellites and betasatellites [4] and pose a serious threat to food and fiber crops throughout the Old World [5].

The DNA A component of Old World begomoviruses encodes six genes; two in the virion-sense (AV1 and AV2) and four in the complementary-sense (AC1, AC2, AC3 and AC4) [6]. However, for some begomoviruses, five complementary-sense genes have also been reported [7]. The virion-sense gene AV2 encodes a pathogenicity determinant that also has a role in virus movement while AV1 encodes the coat protein. The complementary-sense gene AC1 encodes the replication associated protein (Rep) while AC3 enhances virus replication. The AC2 promotes transcription of virion-sense genes and is often involved in overcoming host immune responses mediated by RNA silencing. The DNA B component of bipartite begomoviruses encompasses one complementary-sense gene (BC1) that encodes movement protein (MP), and one virion-sense gene (BV1) encoding the nuclear shuttle protein (NSP) [6]. DNA A and DNA B components share little sequence identity except for a “common region” (CR) of 200–400 bp. The CR includes a predicted stem-loop structure with the nonanucleotide motif TAATATTAC in the loop, which is the origin of replication for geminiviruses [8,9,10]. Begomoviruses have become a major limiting factor in the production of various food and fiber crops such as tomato, cassava, cucurbits, pepper, beans and cotton [11].

Begomoviruses native to the OW are distinct from those native to the New World. Most of the bipartite begomoviruses are native to NW and only a small number occur in the OW. *Watermelon chlorotic stunt* *virus* (WmCSV) is a bipartite begomovirus that was first identified in Yemen in 1988 [12] and then, in subsequent years, reported from across the Middle East and North Africa [7,13]. Symptoms caused by the virus are vein yellowing, chlorotic mottling, stunting and severe reduction of yield, mainly in watermelon (*Citrullus spp.*) crops. However, the host range of the virus extends to most cucurbits including melons, cucumber, pumpkin and squash. Experimental plants such as *Nicotiana benthamiana* and *N. glutinosa* have also been found to be hosts [14]. 

Here we have isolated WmCSV from squash plants grown in an experimental field of Sultan Qaboos University, Oman. The phylogenetic relationship to other WmCSV isolates and infectivity to *N. benthamiana* were assessed. 

## 2. Results and Discussion

### 2.1. Cloning and Sequencing

Leaves of squash plants showing symptoms typical of begomovirus infection were collected from the Agricultural Experimental Station (Sultan Qaboos University, Oman). Total genomic DNA was isolated and rolling-circle amplification (RCA) was used for the amplification of circular DNA molecules [15]. Digestion of the RCA product with *Bam*HI resulted in ~2.7 kb fragments which were cloned into the plasmid vector pUC19. A total seven clones were obtained and sequenced (Macrogen Inc., Korea) using the primer walking strategy. The sequences are available in the nucleotide sequence databases under the accession numbers listed in Table 1.

### 2.2. Sequence Comparisons and Analysis

Comparisons of the sequences obtained, using the ClustalW algorithm implemented in MegAlign (part of the Lasergene package of sequence analysis software; DNAStar Inc., Madison, WI, USA) showed 5 of the sequences to share 99–100% nucleotide sequence identity, indicating that they are isolates of a single species, based on presently applicable species demarcation criteria [16]. Comparisons to sequences available in the databases showed the DNA A sequences obtained here to have the highest levels of sequence identity (96.5–98.8%) to the DNA A components of WmCSV (Table 2). The overall highest levels of identity were to an isolate of WmCSV originating from Iran (AJ245652). This indicates that all 5 sequences obtained from Oman represent isolates of WmCSV.

**Table 1:** Origins of virus isolates and features of the DNA A and DNA B components of *Water melon chlorotic stunt virus* from Oman.

Isolate	Isolate Descriptor	**DNA A**									**DNA B**			
		**Accession Number**	**Size (nt)**	**Position of Genes (Coordinates of Start/Stop Codons [Predicted Coding Capacity in kDa])**							**Accession Number**	**Size (nt)**	**Position of Genes (Coordinates of Start/Stop Codons [Predicted Coding Capacity in kDa])**	
				**AV1**	**AV2**	**AC1**	**AC2**	**AC3**	**AC4**	**AC5**			**BV1**	**BC1**
Als-1	[OM:Squ**1**:11]	JN618981	2752	315–1094 (28.6)	155–514 (13.2)	1540–2625 (39.8)	1233–1640 (15.0)	1088–1492 (14.9)	2328–2471 (5.3)	219–986 (28.2)	JN618980	2744	516–1271 (27.7)	1308–2231 (33.9)
Als-2	[OM:Squ**2**:11]	JN618982	2755	315–1094 (28.6)	155–514 (13.2)	1540–2628 (39.9)	1233–1643 (15.1)	1088–1492 (14.9)	2331–2474 (5.4)	219–986 (28.2)	HE800539	2726	500–1255 (27.7)	1295–2215 (33.8)
Als-3	[OM:Squ**3**:11]	JN618983	2755	315–1094 (28.6)	155–514 (13.2)	1540–2628 (39.9)	1233–1643 (15.1)	1088–1492 (14.9)	2331–2474 (5.4)	219–986 (28.2)	_	_	_	_
Als-4	[OM:Squ**4**:11]	JN618984	2752	315–1094 (28.6)	155–514 (13.2)	1540–2625 (39.8)	1233–1640 (15.0)	1088–1492 (14.9)	2328–2471 (5.3)	219–986 (28.2)	_	_	_	_
Als-5	[OM:Squ**5**:11]	JN624386	2752	315–1091 (28.5)	155–514 (13.2)	1540–2625 (39.8)	1233–1640 (15.0)	1088–1492 (14.9)	2328–2471 (5.3)	219–986 (28.2)	_	_	_	_

**Table 2:** Percentage nucleotide sequence identities of WmCSV clones isolated from Oman with WmCSV isolate sequences available in the databases.

WmCSV Isolates from Oman		WmCSV isolates from the databases * (accession numbers of DNA A/DNA B)						
Component	Isolate	Iran (AJ245652/AJ245653)	Sudan (AJ245650/AJ245651)	Yemen (AJ012081/AJ012082)	Jordan (EU561237/EU561236)	Israel (EF201809/EF201810)	Lebanon (HM368371/HM368372)	Palestine * (JN673223)
DNA A	Als-1	98.8	98.0	96.7	97.7	97.7	97.6	97.9
	Als-2	98.6	97.8	96.6	97.5	97.6	97.5	97.7
	Als-3	98.5	97.7	96.5	97.4	97.5	97.4	97.5
	Als-4	98.8	98.0	96.7	97.7	97.8	97.7	98.0
	Als-5	98.7	98.0	96.6	97.6	97.7	97.6	98.0
DNA B	Als-1	96.9	92.2	93.4	91.9	92.5	91.8	-
	Als-2	97.4	92.5	93.3	93.4	92.9	92.2	-

^*^ No DNA B sequence is available for the Palestine isolates; The sequence with the highest level of identity in each case is underlined.

**Figure 1:**
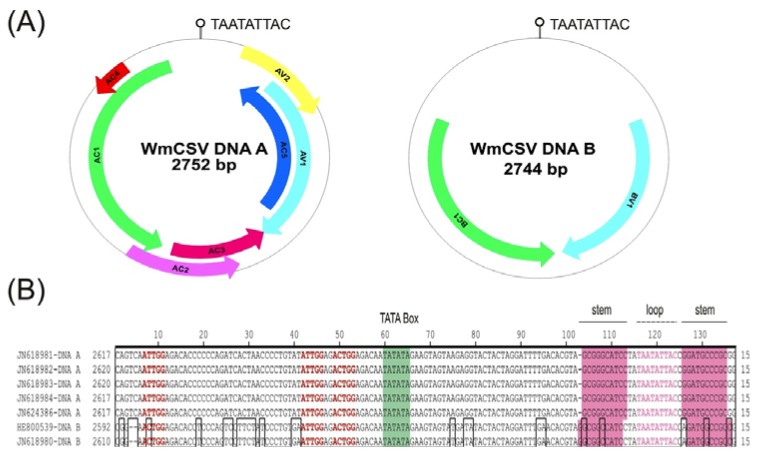
Arrangement of the genomic components of WmCSV (**A**). The positions and orientations of genes are shown with colored arrows. The position of the predicted stem-loop structure (containing the nonanucleotide sequence; TAATATTAC) is indicated. An alignment of the common region sequences of the DNA A and DNA B components of WmCSV isolates from Oman is shown in (**B**). Spaces (-) are introduced to optimize the alignment. Differences in sequences are indicated with boxes. Stem and loop sequences (pink highlighting and pink text, respectively) of the predicted stem-loop structure, position of iterons (red text) and TATA box (highlighted in green) of the Rep promoter are indicated.

A more detailed analysis of the DNA A sequences from Oman showed them to encompass seven open reading frames (ORFs) which are positionally conserved with those of other begomoviruses; two in the virion sense (AV1 and AV2) and the five in complementary sense (AC1 to AC5; Figure 1 (A)). The AC5 ORF is not conserved between all begomoviruses and may not thus be required for infectivity. Inoculation of plants with a WmCSV DNA A clone-with the AC5 mutated, in the presence of the DNA B, induced symptoms indistinguishable from the wild-type, showing that the gene is not required for infectivity and symptom induction [7]. However, for the bipartite begomovirus *Mungbean yellow mosaic virus* (MYMV) studies in yeast have suggested that the product of AC5 may be involved in viral replication [17]. A predicted stem-loop structure, containing the sequence TAATATTAC within the loop, was identified in all sequences. This marks the origin of virion-strand DNA replication for geminiviruses [18,19]. The virion sense genes are highly conserved; the AV2 exhibiting 97–100% amino acid (aa) sequence identity with the AV2 protein of the other WmCSV isolates but less than 81% with the AV2 proteins of other bipartite begomoviruses. The coat protein (CP; encoded by AV1) showed 95–99% identity to other WmCSV isolates but less than 81% identity to the CPs of other begomoviruses. The sequences of the Rep (encoded by AC1) showed 98.1 to 99.2% aa sequence identity to other WmCSV isolates. The replication enhancer protein (REn; encoded by AC3) is involved in regulating the viral level in host plants. Mutation of REn leads to reduced virus titer, but is not essential for the replication of virus [20]. The REn sequences were identical among the WmCSV isolates from Oman but showed 94–98% aa identity to REn proteins of the other isolates. However it shares less than 77% identity to all other begomovirus REn proteins in the databases. The AC2, which overlaps AC3 encodes the transcriptional activator protein (TrAP) involved in the activation of late genes [21]. The TrAP sequences of the Oman isolates share 99–100% identity, while the range is 93 to 99% identity to all other WmCSV isolates. TrAP proteins of begomoviruses other than WmCSV show less than 65% sequence identity with the TrAP of the Oman isolates. AC4 is a highly conserved small protein contained entirely within the Rep sequence but in a different reading frame [22]. The AC4 aa sequences of the Oman isolates are identical to each other and share 98% identity with other WmCSV isolates, but less than 72% identity to the AC4 sequences of other begomoviruses in the databases. 

Two clones of DNA B (JN618980 and HE800539) were obtained with a size of 2744 and 2726 bp, respectively. The sequences contain two ORFs, as found in the DNA B of all bipartite begomoviruses; BC1 (encoding the movement protein) in the complementary-sense and BV1 (encoding the nuclear shuttle protein) in the virion-sense. These proteins are involved in intracellular and cell to cell movement of the virus in plants [23,24].

**Figure 2:**
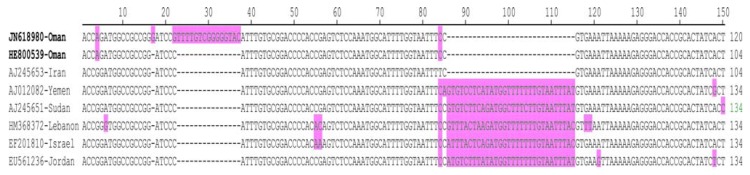
Alignment of CR sequences of the DNA B components of WmCSV from Oman and the database sequences from different origins. Spaces (-) are introduced to optimize the alignment. Differences in the sequences are highlighted. Oman isolates are marked as bold.

The two DNA B clones from Oman share 99% sequence identity to each other and the highest level of identity (96.9 and 97.4%, respectively) to the Iran DNA B isolate. All other WmCSV DNA B sequences in the databases the levels of identity were less than 93.4% (Table 2). An alignment of DNA B sequences shows both isolates from Oman to lack a stretch of 30 bp (coordinates 85 to 115), in the CR downstream of the hairpin structure, in comparison to all other WmCSV DNA B sequences except that from Iran (Figure 2). This deletion, distinguishing the Iran isolate from all other WmCSV isolates, was first identified by Abudy *et al*. [25]. Additionally, the DNA B clone of isolate Als-1 (JN618980) contains a 15 bp insertion (coordinates 22 to 37) which distinguishes it from all other WmCSV DNA B sequences, including that of isolate Als-2. Overall the sequences of the DNA B from Oman and Iran have high levels of sequence identity and are distinct from the sequences of the other isolates. These findings strongly suggest that WmCSV present in Oman was introduced from Iran.

**Figure 3:**
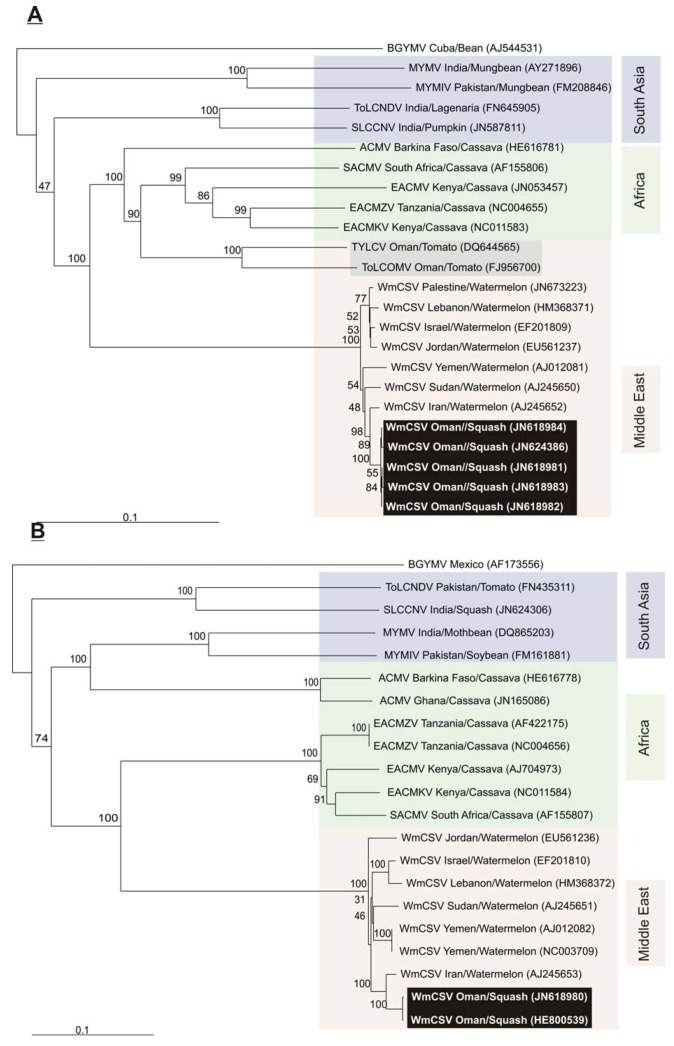
Neighbor joining phylogenetic dendrogram based upon alignments of the sequences of the genomes for DNA A components (**A**) and DNA B components (**B**) of WmCSV and selected other begomoviruses from the databases. In each case the database accession number is given. The numbers at nodes represent percentage bootstrap confidence scores (1000 replicates). The alignment was arbitrarily rooted on outgroup *Bean golden yellow mosaic virus* (BGYMV), a distantly related begomovirus. Begomovirus acronyms used are *Mungbean yellow mosaic virus* (MYMV), *Mungbean yellow mosaic India virus* (MYMIV), *Tomato leaf curl New Delhi virus* (ToLCNDV), *Squash leaf curl China virus* (SLCCNV), *African cassava mosaic virus* (ACMV), *South African cassava mosaic virus* (SACMV), *East African cassava mosaic virus* (EACMV), *East African cassava mosaic Zanzibar virus* (EACMZV), *East African cassava mosaic Kenya virus* (EACMKV), *Tomato yellow leaf curl virus* (TYLCV) and *Tomato leaf curl Oman virus * (ToLCOMV). The isolates characterized here are indicated with white text on black background. Other begomoviruses from Oman are highlighted by a gray background. Additionally the begomoviruses originating from the Middle East, Africa and southern Asia are indicated by colored boxes.

An alignment of the CR of WmCSV DNA A and DNA B upstream of the hairpin structure, showing the sequence of the predicted stem loop structure, the TATA box of the Rep promoter and the predicted iterons (the sequences which are recognized by the Rep to start virion-strand replication of the genome) is shown in Figure 1 (B) [26,27,28]. The CR of DNA A and DNA B is 140 bp in length shares 76.3% sequence identity. This high identity percentage suggests that the two DNA components are cognate. Phylogenetic analysis of the sequences obtained here shows them to group with previously reported isolates of WmCSV (Figure 3). For both the DNA A and DNA B components the sequences segregate most closely with the sequences of a WmCSV isolate from Iran, suggesting its probable route of entry from Iran.

### 2.3. Infectivity of WmCSV

**Figure 4:**
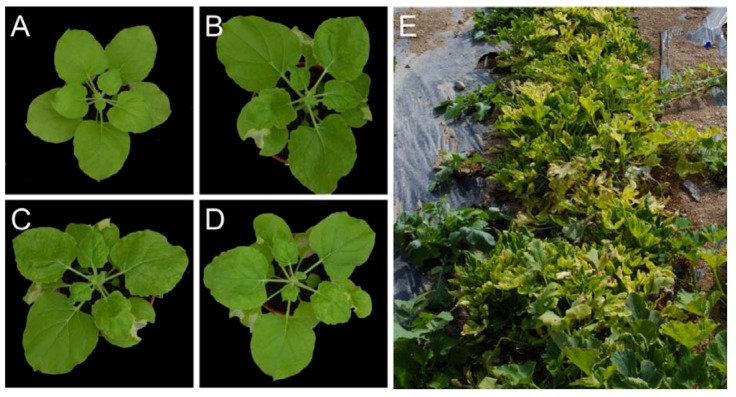
Symptoms of plants infected with WmCSV. A non-inoculated healthy *N. benthamiana* plant (**A**); *N. benthamiana* plants inoculated with the clones of WmCSV (**B–D**) and squash plants in the field infected with WmCSV (**E**).

Partial repeat constructs of both the components of WmCSV were co-inoculated using *Agrobacterium tumefaciens*. The plants began to show mild symptoms of infection at 10 days post inoculation (dpi), exhibiting leaf crumpling symptoms at 15 dpi. However, the symptoms persisted for only one month and then started to diminish. This “recovery” of the plants is likely due to RNA silencing [29]. Severe necrosis was also observed in the inoculated patches of leaves as shown in Figure 4 ((B), (C) and (D)) which means that *N. benthamiana* has resistance to this bipartite begomovirus; another possible explanation for the development of mild symptoms and recovery. Systemic movement of virus was confirmed by diagnostic PCR using primers designed to amplify the CP genes of the majority of begomoviruses.

Oman has extensive agricultural trading links with Iran and the highly destructive begomovirus *Tomato yellow leaf curl virus* present in Oman is believed to have its origins in Iran [30]. Introduction of geminiviruses and associated satellites from India/Pakistan and Singapore [31] and southern Africa (A.J. Khan, manuscript in preparation) into Oman could possibly be attributed to the wider trade links and air traffic between Oman and other countries in this region. This confluence of viruses and satellites from such widely separated geographic areas is of great concern due to the possibility of new viruses arising by recombination or by new interactions between viruses and satellites arising. Examples of both these possibilities have already been reported [30,31]. The identification of WmCSV in Oman indicates that the diversity of viruses here is greater than previously known and adds to the worry that, due to the extensive trade ties (particularly air travel), the viruses may spread outwards to other parts of the world. 

## 3. Experimental Section

### 3.1. Sample Collection and DNA Extraction

In the summer of 2011, squash (*Cucurbita moschata*) plants growing in the experimental fields of Agricultural Experimental Station (Sultan Qaboos University, Oman) showed curling, chlorosis and stunted growth, reminiscent of begomovirus infection (Figure 3 (E)). Leaves were collected from symptomatic squash plants and genomic DNA was extracted using the CTAB method [32].

### 3.2. Amplification, Cloning and Sequencing of Virus Components

The extracted DNA was used as a template for rolling circle amplification (RCA) [33] using the TempliPhi 100 Amplification kit (Amersham Biosciences, Piscataway, NJ, USA) following the manufacturer’s instructions. For each RCA reaction, a total reaction mixture of 11.2 µL was prepared that consisted of 1 µL of genomic DNA as template, 5 µL of sample buffer (containing random hexamer primers), 5 µL of reaction buffer and 0.2 µL of enzyme mix (φ29 DNA polymerase and pyrophosphatase). 1 µL of template DNA was mixed with 5 µL of sample buffer and heated at 95 °C for 3 minutes to denature the DNA and primers. During the denaturation step, a reaction premix of 5 µL reaction buffer and 0.2 µL enzyme mix for each sample was prepared. Once the tubes had cooled from denaturation temperature, 5 µL of the freshly made premix was added to each sample and the resulting reaction mixtures were incubated at 30 °C for 18 hours. Resulting high molecular weight products were then digested with a range of restriction endonucleases to identify enzymes giving unit length (either 2.8 or 1.4 kb) fragments, which were cloned into the plasmid vector pUC19 and sequenced (Macrogen Inc., Korea). 

### 3.3. Sequence Analysis

Sequences were stored and analyzed using the Lasergene package of sequence analysis software (DNAStar Inc., Madison, WI, USA). Open reading frames were predicted using ORF Finder run online (NCBI). Sequence similarity searches were performed by comparing the sequence to other begomovirus sequences available in the GenBank database using BLAST. Multiple sequence alignments were performed using MegAlign (Lasergene) and ClustalX [34]. Phylogenetic trees were constructed using the neighbor joining algorithm of ClustalX and displayed, manipulated and printed using TreeView [35].

### 3.4. Agrobacterium-Mediated Inoculation

A partial direct repeat construct of the DNA A component of WmCSV (JN618981) was produced by digestion with *Bam*HI and *Eco*RI to release an approximately 1.3 kb fragment which was ligated into the binary vector pCambia1301. This partial clone was then linearized with *Bam*HI and the full-length DNA A component, released with *Bam*HI was ligated into the vector to yield a partial direct repeat of the component. Similarly a partial dimer construct of the DNA B of WmCSV (JN618980) was also produced for *Agrobacterium*-mediated inoculation. The clone SQY-26 was digested with *Bam*HI and *Pst*I to release an approximately 1.9 kb fragment which was ligated into the binary vector pCambia1301. The partial clone of pCambia1301 was then digested with *Bam*HI and the full-length component was inserted.

The binary vector constructs were transferred into *Agrobacterium tumefaciens* strain LBA4404 by electroporation. A single bacterial colony was picked from the plate using sterile wire loop and transferred to 50 mL LB medium containing antibiotic selection and incubated at 28 °C with a shaking of 150 rotations per minute for 48 hours until an O.D.600 of 1 was attained. The cells were harvested by centrifugation and resuspended in 10 mM MgCl_2_ containing 100 µM acetosyringone. This inoculum was infiltrated into the undersides of the leaves of *Nicotiana benthamiana* plants (at the 6 to 8 leaf stage) using a 5 mL syringe. The plants were kept at 26 °C in a glasshouse with supplementary lighting to give a 16 h day length.

### 3.5. Diagnostic PCR

Diagnostic PCR of inoculated plants was conducted using degenerate primers (unpublished data) designed on the CP region of begomoviruses to get an amplification product of 650 bp. PCR profile used in thermocycler was; initial denaturation at 95 °C for 3 minutes, 35 cycles were repeated with denaturation at 94 °C for 1 minute, annealing at 52 °C for 2 minutes and extension at 72 °C for 2 minutes. Final extension was provided for 10 minutes. Amplified products were visualized in 1% agarose gels (stained with ethidium bromide) under ultraviolet light. 

## 4. Conclusions

This is the first report on the occurrence of WmCSV in Oman. The sequence of WmCSV from Oman shows the highest levels of identity to a WmCSV isolate from Iran, which indicates that the virus likely has been introduced from Iran. Squash, melons and cucumber are important crops in Oman, which are increasingly affected by this virus. The presence of WmCSV provides the possibility of recombination with other prevalent begomoviruses, which may lead to evolution of new and more aggressive viruses. This region of the Middle East has extensive trade/travel links with the rest of the world, so the chances of this virus spreading are high. 
